# Comparison of Visual Outcome and Morphologic Change between Different Surgical Techniques in Idiopathic Epiretinal Membrane Surgery

**DOI:** 10.1155/2018/4595062

**Published:** 2018-04-16

**Authors:** Yo-Chen Chang, Chia-Ling Lee, Kuo-Jen Chen, Li-Yi Chiu, Tzu-En Kao, Pei-Kang Liu, Kwou-Yeung Wu, Wen-Chuan Wu

**Affiliations:** ^1^Department of Ophthalmology, Kaohsiung Medical University Hospital, Kaohsiung 80708, Taiwan; ^2^Department of Ophthalmology, School of Medicine, Kaohsiung Medical University, Kaohsiung 80708, Taiwan; ^3^Department of Ophthalmology, Kaohsiung Municipal Hsiao-Kang Hospital, Kaohsiung Medical University, Kaohsiung 81267, Taiwan; ^4^Department of Ophthalmology, Yuan's General Hospital, Kaohsiung, Taiwan

## Abstract

**Purpose:**

To investigate the morphological and functional outcomes of idiopathic epiretinal membrane (ERM) surgery between three different surgical techniques: ERM peeling only, whole-piece ILM peeling, and maculorrhexis ILM peeling.

**Patients and Methods:**

This is a retrospective, consecutive, and comparative study enrolling 60 patients from Kaohsiung Medical University Hospital, Kaohsiung, Taiwan. Surgery performed between July 2011 and June 2012 was done with ERM peeling only (group I). ERM peeling and ILM peeling as a whole piece (group II) were performed between July 2012 and July 2013. Surgery performed between August 2013 and December 2014 was done with maculorrhexis ILM peeling (group III). Main outcome measures include visual acuity change (BCVA) and central foveal thickness (CFT).

**Results:**

At 12 months postoperation, the mean BCVA in group III was significantly better than in group I and group II. Comparison of CFT reduction between the three groups revealed significantly more reduction in group III than in group II at all postoperative follow-up periods. Eyes with restoration of foveal depression were observed in 52.6% in group I, 52.4% in group III, but only 20% of eyes in group II. None of the eyes in both ILM peeling groups encountered recurrence of macular pucker formation.

**Conclusion:**

All three techniques can achieve visual acuity improvement and macular thickness reduction. Maculorrhexis ILM peeling achieves more rapid improvement of visual function, better final visual outcome, and a higher rate of normal foveal contour than whole-piece ILM peeling.

## 1. Introduction

Idiopathic epiretinal membrane (ERM) is a disorder occurring in the vitreomacular interface that can cause visual impairment [1]. The clinical manifestation of an ERM can be completely asymptomatic or profoundly symptomatic with metamorphopsia, micropsia or macropsia, decreased visual acuity (VA), and loss of central vision. In 1978, Machemer first applied pars plana vitrectomy (PPV) and membrane peeling to remove ERM. From now on, it has become a well-established procedure for the removal of ERM with good results [2]. Surgical removal of the membranes in symptomatic patients can reduce metamorphopsia and improve visual acuity in approximately 70–90% of cases [3–5]. However, the recurrence of ERM has been reported in 10% to 21% of eyes with membrane peeling [6–8]. The possible pathogenesis of ERM regrowth is thought to be due to incomplete ERM removal and the presence of residual myofibroblasts [9, 10]. Recently, in order to prevent the recurrence of ERM, internal limiting membrane (ILM) peeling has been applied in ERM surgery. Compared with ERM peeling only, conventional whole-piece ILM peeling after ERM peeling can achieve comparable visual improvement and reduced ERM recurrence [11, 12]. However, remaining thickened macula postoperatively and formation of postoperative central or eccentric macular hole have been reported [11, 13]. In order to prevent the above complications, we used a modification of ILM peeling technique named “maculorrhexis ILM peeling” to improve the surgical outcome for patients with ERM.

## 2. Patients and Methods

**Figure 1:**
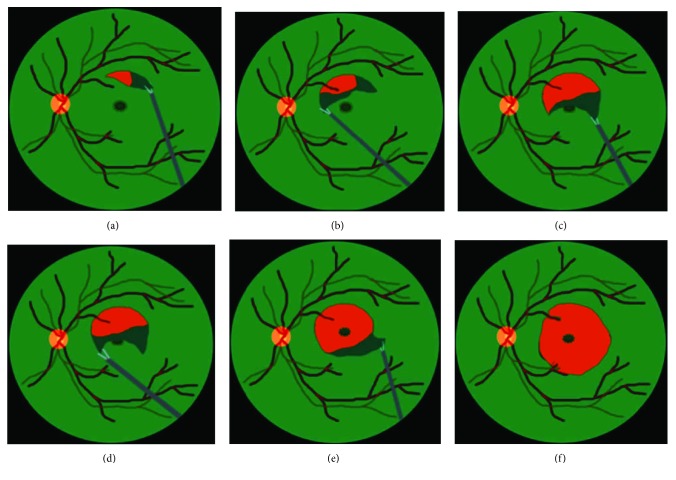
Schematic drawing of whole-piece internal limiting membrane (ILM) peeling. (a) After indocyanine green (ICG) staining, first create an ILM flap with 25-gauge forceps near the vascular arcade. (b, c) Expand the flap from both sides. (d, e) Peel off the ILM as a large whole piece passing through the fovea. (f) Constantly adjust the force to keep the flap large enough and prevent its immature rupture near the fovea.

**Figure 2:**
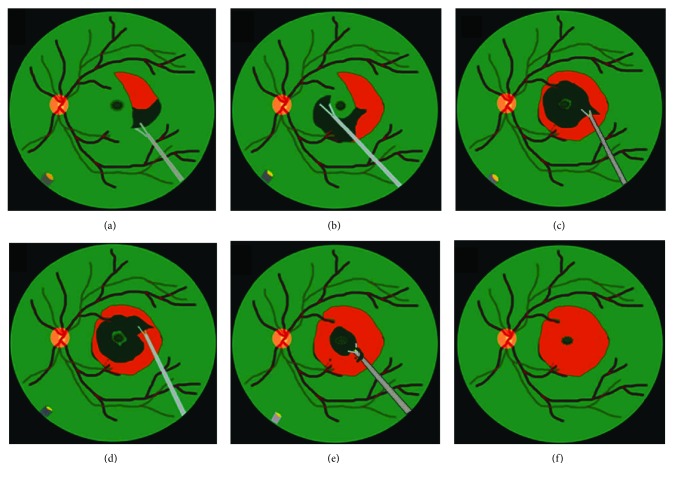
Schematic drawing of maculorrhexis ILM peeling. (a) After ICG staining, first create an ILM flap with 25-gauge forceps near the vascular arcade. (b) Proceed with ILM peeling in a circular fashion, caution must be taken not to peel off the central foveal area. (c, d, e) After finishing peeling of peripheral round of ILM, paracentral ILM was also gently peeled in a circular fashion. (f) When the circle is nearly complete, the ILM on the fovea is peeled off gently.

The present study is a retrospective, consecutive case series. Between January 2012 and December 2014, we enrolled patients who were diagnosed as idiopathic ERM. The research adhered to the tenets of the Declaration of Helsinki 1964. Spectral-domain optical coherence tomography (SD-OCT; Heidelberg Retina Angiograph 2, Heidelberg Engineering, Heidelberg, Germany) was used to confirm the presence of ERM. Patients with the history of ocular diseases (i.e., retinal vascular occlusion, high myopia, glaucoma, neoplastic, or chronic inflammatory disorders), cases with spontaneous peeling of ILM during ERM peeling, and those with systemic diseases (uncontrolled hypertension or diabetes) were excluded. Preoperatively, a complete ophthalmic and medical history was obtained, and a detailed examination including best-corrected visual acuity (BCVA) measured by Snellen chart, intraocular pressure, fundus examination by fundus photography, indirect binocular ophthalmoscopy, and SD-OCT was performed. All patients underwent 25-gauge PPV and epiretinal membrane peeling assisted with triamcinolone and high-magnification viewing system. If the posterior vitreous detachment (PVD) was not already present, it was induced by active suction of ocutome above the optic disc. Surgeries before July 2012 were done with PPV and ERM peeling only. Indocyanine green- (ICG-) assisted ILM peeling as a whole piece was performed after ERM peeling between July 2012 and July 2013 (Figure 1). Surgeries after July 2013 were performed with the newly developed maculorrhexis ILM peeling technique (Figure 2) [14]. For eyes undergoing ILM peeling, an intravitreal injection with indocyanine green, which was mixed as per the bottle instructions with sterile water then diluted in a 1 : 24 ratio with 5% glucose water, was performed to make the internal limiting membrane (ILM) more visible. Concomitant cataract surgery was performed on phakic patients. After surgery, comprehensive ophthalmic examination including SD-OCT was performed 1, 3, 6, 9, and 12 months postoperatively. For better comparison of visual outcome between the groups, the visual acuity measured at preoperative and each postoperative follow-up visit was converted to the logarithm of the minimum angle of resolution (logMAR).

### 2.1. Statistical Analysis

All data were analyzed by the Fisher's exact test and Student's *t*-test, using SPSS statistical software (version 13.0; SPSS Inc., Chicago, IL, USA). A difference at *p* < 0.05 was considered to be statistically significant.

## 3. Results

### 3.1. Preoperative Demographic Data

A total of 60 eyes in 60 patients were included in the present study. The mean age was 64.4 ± 7.6 years. There were 19 eyes that underwent ERM removal only (group I), 20 eyes underwent ERM removal and whole-piece ILM peeling (group II), and 21 eyes underwent ERM removal with maculorrhexis ILM peeling (group III). Eight eyes in group I, 8 eyes in group II, and 10 eyes in group III were phakic prior to surgery. The mean preoperative logMAR BCVA and central foveal thickness (CFT) for the total 60 eyes were 0.79 ± 0.42 and 491.5 ± 114.8 *μ*m, respectively. The major characteristics for these three groups were similar and without significant difference. The patient details for group I, group II, and group III are listed in Table 1.

### 3.2. Temporal Change of CFT

**Figure 3:**
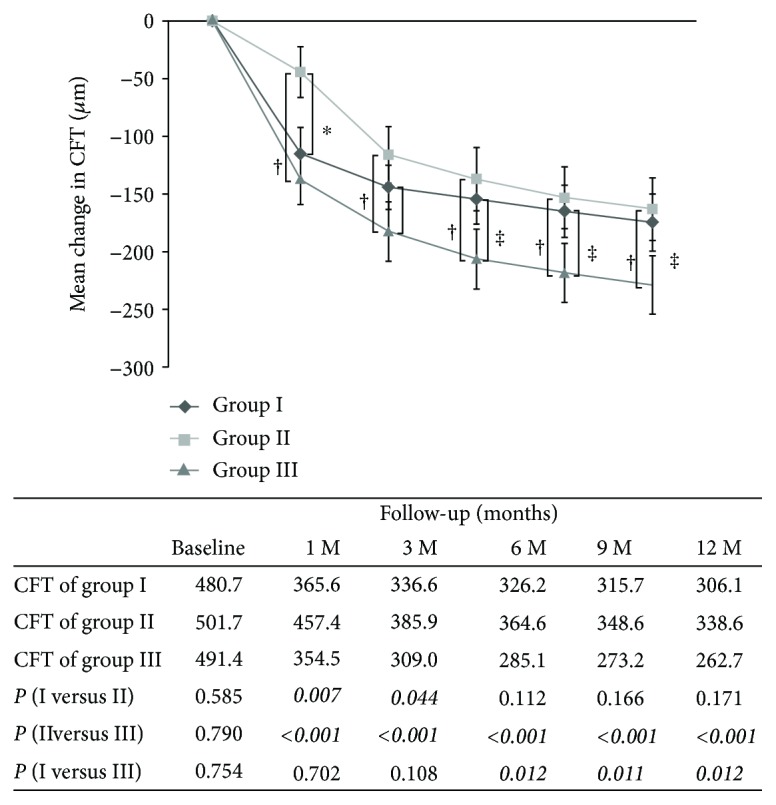
Temporal change of central foveal thickness (CFT) postoperatively. The reduction in CFT differed significantly between group I and group II during the first month after surgery. The reduction in CFT differed significantly between group II and group III at 1, 3, 6, 9, and 12 months of follow-up. The table below the graph shows the absolute values of the mean CFT for each follow-up visit (group I: ERM removal without ILM peeling; group II: ERM removal and whole-piece ILM peeling; group III: ERM removal and maculorrhexis ILM peeling; ∗ indicates *p* < 0.05 compared between group I and group II at each follow-up visit; † indicates *p* < 0.05 compared between group II and group III at each follow-up visit; ‡ indicates *p* < 0.05 compared between group I and group III at each follow-up visit).

The mean preoperative CFT was 480.7 ± 102.6 *μ*m for group I, 501.7 ± 132.7 *μ*m for group II, and 491.4 ± 111.6 *μ*m for group III (Table 1). Significant differences in CFT were not found between these three groups. The mean CFT at 1 month after surgery decreased by 115.1 ± 99.8 to 365.6 ± 113.3 *μ*m for group I, by only 44.3 ± 98.3 to 457.4 ± 86 *μ*m for group II, and by 137.0 ± 100.4 to 354.5 ± 64.8 *μ*m for group III, (*p* = 0.007, group I versus group II; *p* < 0.001, group II versus group III; *p* = 0.7, group I versus group III). At 6 months, the mean CFT decreased by 154.5 ± 93.9 to 326.2 ± 63.3 *μ*m for group I, by 137.1 ± 112.4 to 364.6 ± 82.2 *μ*m for group II, and by 206.3 ± 118.6 to285.1 ± 32.0 *μ*m for group III (*p* = 0.112, group I versus group II; *p* < 0.001 group II versus group III; *p* = 0.012 group I versus group III). At 12 months, the mean CFT decreased by 174.6 ± 107.7 to 306.1 ± 71.5 *μ*m for group I, by 163.1 ± 121.1 to 338.6 ± 73.8 *μ*m for group II, and by 228.7 ± 115.9 to 262.7 ± 25.7 *μ*m for group III (*p* = 0.171, group I versus group II; *p* < 0.001, group II versus group III; *p* = 0.013, group I versus group III). The CFT differed significantly between group I and group II at 1 and 3 months after surgery (*p* < 0.05). The mean CFT was significantly higher in group II than it was in group III at 1, 3, 6, 9, and 12 months of follow-up. The mean change in CFT and mean CFT over the course after surgery was shown in Figure 3.

### 3.3. Temporal Change of Visual Acuity

**Figure 4:**
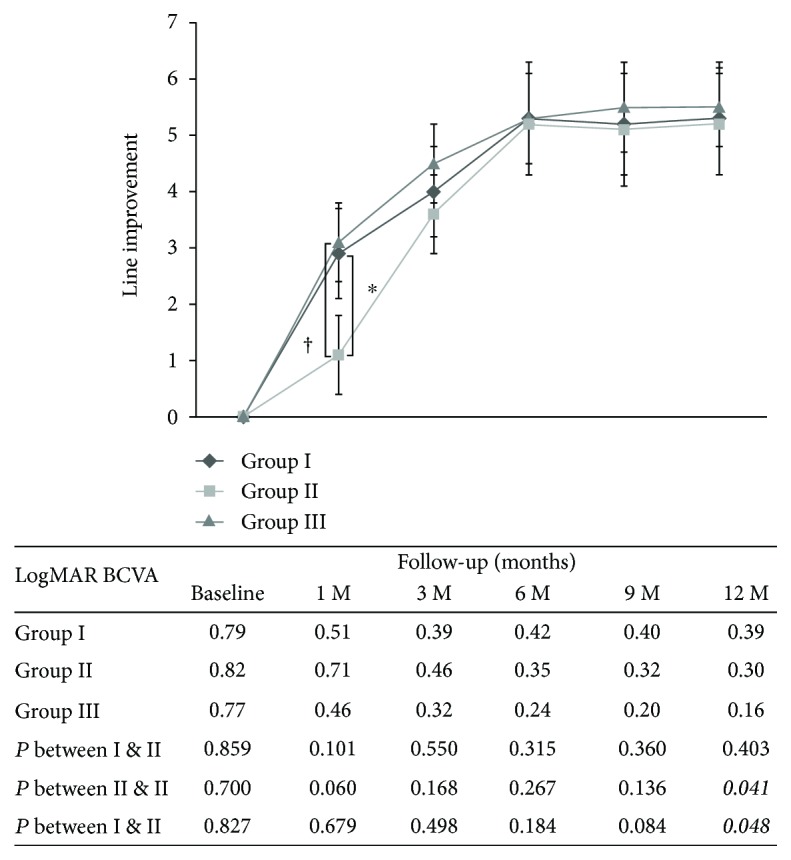
Temporal change of BCVA by line after surgery. At 1 month, the mean line improvement increased more rapidly in group I and group III than in group II. However, 6 months and thereafter, the postoperative line improvement and BCVA were similar and became stable in all three groups. The table below the graph shows the absolute value of the mean logMAR BCVA for each follow-up visit (Group I: ERM removal without ILM peeling; Group II: ERM removal and whole-piece ILM peeling; Group III: ERM removal with maculorrhexis ILM peeling; ∗ indicates *p* < 0.05 compared between group I and group II at each follow-up visit; † indicates *p* < 0.05 compared between group II and group III at each follow-up visit).

Before surgery, the mean BCVA was 0.79 ± 0.40, 0.82 ± 0.48, and 0.77 ± 0.39 logMAR in group I, group II, and group III, respectively. The preoperative BCVA was similar, and no significant difference between these three patient groups was observed (Table 1). In all three groups, the BCVA improved significantly postoperatively. At 1 month, the line improvement increased by 2.9 ± 3.4, 1.1 ± 3.2, and 3.1 ± 3.0 lines from preoperation in group I, group II, and group III, respectively (*p* = 0.094, group I versus group II; *p* = 0.841, group I versus group III; *p* = 0.049, group II versus group III). At 6 months, the mean line improvement increased by 3.8 ± 4.0, 4.8 ± 3.8, and 5.3 ± 3.6 lines from preoperation in group I, group II, and group III, respectively. At 12 months, the mean BCVA was increased by 4.0 ± 4.3, 5.2 ± 4.2, and 6.1 ± 3.3 lines from preoperation in group I, group II, and group III, respectively. At 12 months, the mean logMAR BCVA improved to 0.39 ± 0.43, 0.30 ± 0.13, and 0.16 ± 0.27 in group I, group II, and group III, respectively.(*p* = 0.403, group I versus group II; *p* = 0.048, group I versus group III; *p* = 0.041, group II versus group III). The logMAR BCVA in group III was significantly better than that in group I and group II at 12 months postoperatively.  Figure 4 illustrates the line improvement in BCVA and mean logMAR BCVA of these three groups over the course of the study.

### 3.4. Morphology of Fovea and Recurrence of Epiretinal Membrane

**Figure 5:**
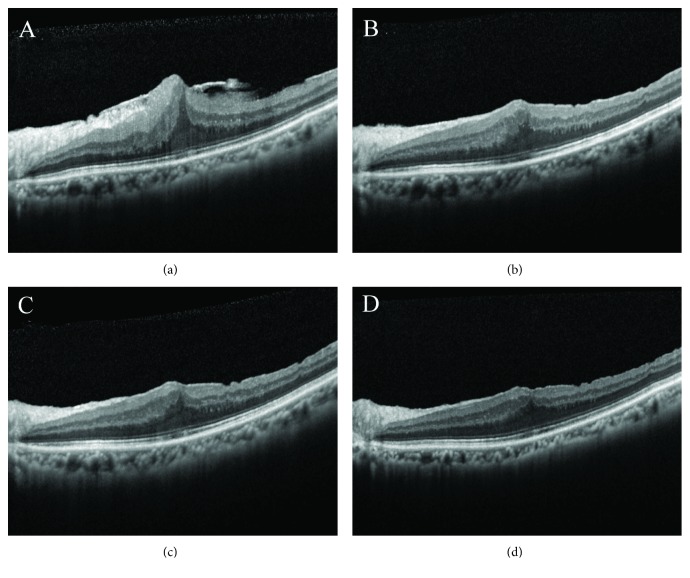
Pre- and postoperative OCT findings in a patient with ERM receiving vitrectomy, membrane peeling, and whole-piece internal limiting membrane peeling. Preoperative image shows an ERM overlying the macula. The CFT and BCVA were 659 *μ*m and 20/100, respectively (a). The CFT decreased to 484 *μ*m one month postoperatively (b). At six months after operation, the CFT was 422 *μ*m (c). At 12 months after operation, the CFT decreased to 382 *μ*m and the BCVA improved to 20/30. However, foveal depression was not observed (d).

**Figure 6:**
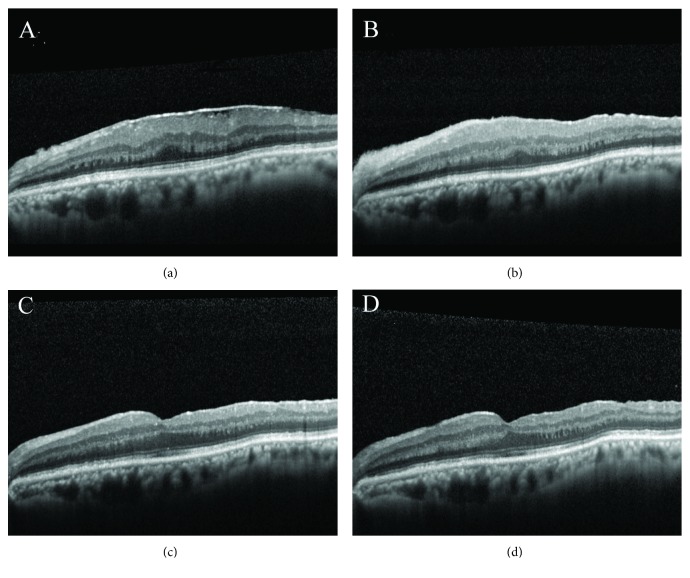
Pre- and postoperative OCT findings in a patient with ERM receiving vitrectomy, membrane peeling, and maculorrhexis ILM peeling. Preoperative image shows an ERM overlying the macula. The CFT and BCVA were 448 *μ*m and 20/80, respectively (a). The CFT decreased to 425 *μ*m one month postoperatively (b). At six months after operation, the CFT was 306 *μ*m and foveal depression was observed (c). At 12 months after operation, the foveal depression still remained. The CFT further decreased to 289 *μ*m, and the BCVA improved to 20/20 (d).

We defined a normal foveal contour on OCT as the retinal thickness at the center of the fovea was 50 *μ*m or more thinner than that of the retina 1 mm away from the foveola, accompanied by a foveal depression without evident intraretinal edema [11]. At 12 months postoperation, normal foveal contour with a foveal depression was found in 10 eyes (52.6%) in group I, only 4 eyes (20%) in group II, and 11 eyes (52.4%) in group III (Table 2). Two representative cases are illustrated in Figures 5 and 6. A dissociated optic nerve fiber layer (DONFL) appearance is defined as arcuate retinal striae along the optic nerve fibers in the macular region, which is slightly darker than the surrounding retina [15]. Postoperative occurrence of DONFL was found in none of the eyes in group I, 10 eyes (50%) in group II, and 7 eyes (33.3%) in group III (Table 2). The external limiting membrane (ELM) and ellipsoid zone (EZ) were evaluated in all three groups. In the preoperative assessment, preservation of ELM was observed in 11 eyes (57.9%) in group I, 13 eyes (65%) in group II, and 13 eyes (61.9%) in group III, respectively. At one month postoperatively, numbers of eyes with intact ELM were still 11 eyes (57.9%) in group I, slightly decreased to 10 eyes in group II, and still 13 eyes (61.9%) in group III. Thereafter, the numbers of eyes with intact ELM continued to increase. At 6 months, preservation of ELM was observed in 19 eyes (100%) in group I, 18 eyes (90%) in group II, and 21 eyes (100%) in group III, respectively. All eyes in group II with intact ELM were observed at 9 months postoperatively (Table 3). With regard to EZ, 12 eyes (63.2%) in group I, 13 eyes (65%) in group II, and 14 eyes (66.7%) in group III showed a normal EZ preoperatively. At 1 month after surgery, still 12 eyes (63.2%) in group I, slightly decreased to 10 eyes (50%) in group II, and still 14 eyes (66.7%) in group III showed intact EZ. At 9 months, preservation of ELM was observed in 19 eyes (100%) in group I, 18 eyes (90%) in group II, and 21 eyes (100%) in group III, respectively. At 12 months, preservation of EZ was observed in all eyes in these three groups (Table 3). ERM recurrence is defined as OCT-based evidence of macular pucker formation. Using this definition, a recurrence within 12 months following surgery was found in 4/19 eyes (21.1%) in group I. However, none of the eyes in group II or group III showed evidence of recurrent ERM during the follow-up period (Table 2).

## 4. Discussion

For patients with symptomatic ERM, pars plana vitrectomy with membrane peeling is a useful technique, and favorable outcome can be achieved in most patients [2–6]. Nevertheless, the recurrence of ERM has been reported in 10% to 21% of eyes with membrane peeling [6–8]. In recent years, conventional whole-piece ILM peeling after ERM peeling can achieve comparable visual improvement and reduced ERM recurrence [11, 12]. Nevertheless, remaining thickened macula postoperatively has been reported [11, 13].

According to our results, the CFT decreased significantly after surgery in all three groups. In the early postoperative period, the CFT decreased relatively slowly and the mean CFT was significantly higher in eyes undergoing whole-piece ILM peeling compared to eyes undergoing maculorrhexis ILM peeling. Furthermore, postoperative OCT showed that loss of the normal foveal contour with macular thickening was more frequently seen in the whole-piece ILM peeling group than it was in the maculorrhexis ILM peeling group and ERM peeling-alone group. The reason for this finding in the present study remains unclear. The possible explanation may be due to the impact on Müller cells by different ILM peeling methods. The outer portion of the ILM is built by the Müller cell footplates. During ILM peeling, the Müller cell footplates might suffer some degree of damage. In addition, in the foveola, the specialized Müller cell formed an inverted cone-shaped zone that constitutes the base of the fovea, serves as a plug that binds the photoreceptor cells, and gives support for the structure [16, 17]. Furthermore, the Müller cells also maintain the arrangement of nerve fiber bundles being close to each other [18]. Therefore, ILM peeling may cause loss of structural support in the fovea and may lead to damage to the nerve fibers. In order to reduce damage to the retina, especially to the Müller cells, we developed maculorrhexis ILM peeling where the ILM is grasped away from the central fovea and peeled off in a circular fashion which is parallel to the arrangement of nerve fiber bundle. We believe that by using this method, the shearing on Müller cells can be decreased and therefore remodeling of the intraretinal structure might be facilitated.

Many investigators have made a lot of efforts to determine etiologies of macular DONFL after ILM peeling. In the present study, postoperative DONFL was observed in none of the eyes in group I, 10 eyes (50%) in group II, and 7 eyes (33.3%) in group III. Our results suggest that this characteristic appearance was related to ILM peeling itself. Ito et al. reported that DONFL occurred in 50% of eyes with ILM peeling and they found that no functional abnormalities were observed, suggesting that DONFL was probably due to a dehiscence of the nerve fiber layer rather than a true defect of nerve fiber [19].

In our study, the integrity of the outer retina was assessed by SD-OCT. In the first month, compared to preoperative data, the number of eyes with intact ELM and EZ was decreased in group II while the number of eyes with intact ELM and EZ remained static in group I and group III. However, these two layers recovered over time and were fully recovered in all patients of three groups after 12 months. De Novelli et al. found that in the first month, in the group that had the ILM removed, there was an increase in the discontinuity of the EZ. They postulated that conventional removal of the ILM may cause additional surgical trauma [20]. However, by using maculorrhexis ILM peeling method, the number of eyes with intact ELM and EZ wasn't decreased at one month postoperatively. The reason for this finding maybe due to the ILM peeling method of circular fashion might place less damaging tension on retina.

In the present study, the recurrence of ERM is significantly lower in patients undergoing ILM peeling compared with those without ILM peeling. From literature review, recurrence of ERM ranges from 10% to 56% after the removal of ERM alone and from 0% to 9% after peeling of both the ERM and ILM [8, 21–23]. When the ILM persists after ERM peeling, this residual ILM acts as a scaffold for cell proliferation leading to ERM recurrence [24]. Therefore, we believe that ILM peeling could not only eliminate residual ERM but also remove the scaffold allowing proliferation of myofibroblasts.

According to our present results, there were significant improvements of BCVA in the ERM peeling only (group I), whole-piece ILM peeling (group II), and the maculorrhexis ILM peeling (group III) groups after ERM surgery. One month postoperatively, the line improvement of BCVA in group I and group III was similar with 2.9 and 3.1 lines, respectively. Both groups were superior to only 1.1 line improvement in group II. We postulate that the early rapid visual improvement in eyes undergoing maculorrhexis ILM peeling might be due to fewer insults to retinal structure. At 12 months postoperation, the BCVA in group III was significantly better than in group I and group II. Previous studies also showed both ERM peeling only and ILM peeling can achieve significant and similar visual improvement postoperatively [22, 25, 26]. Our results were consistent with theirs. However, even better visual outcome can be achieved by using maculorrhexis ILM peeling method.

In summary, our preliminary data of combined 25-gauge vitrectomy, ERM peeling, and ICG-assisted maculorrhexis ILM peeling showed relatively rapid improvement of visual function, better final visual outcome, and normal foveal contour and avoid recurrence of macular pucker in patients with idiopathic ERM. The limitation of our study included relative small number of patients and no randomization. Therefore, these results had to be confirmed by a large prospective and randomized trial.

## Figures and Tables

**Table 1:** Preoperative demographics of patients in three groups.

	Total (*n* = 60)	Group I (*n* = 19)	Group II (*n* = 20)	Group III (*n* = 21)	*p* I versus II	*p* II versus III	*p* I versus III
Age (yrs)	64.4 ± 7.6	65.7 ± 7.4	64.0 ± 8.1	63.4 ± 7.4	0.489	0.815	0.329
M/F	25/35	8/11	9/11	8/13	1.000	0.756	1.000
Phakia	26 (43.3%)	8 (42.1%)	8 (40%)	10 (47.6%)	1.000	0.756	0.761
LogMAR	0.79 ± 0.42	0.80 ± 0.40	0.82 ± 0.48	0.77 ± 0.40	0.859	0.700	0.827
CFT (*μ*m)	491.5 ± 114.8	480.1 ± 102.6	501.7 ± 13.27	491.4 ± 111.6	0.585	0.790	0.754

CFT: central foveal thickness; ERM: epiretinal membrane; F: female; Group I: ERM removal without ILM peeling; Group II: ERM removal and whole-piece ILM peeling; Group III: ERM removal and maculorrhexis ILM peeling; ILM: internal limiting membrane; LogMAR: logarithm of the minimum angle of resolution; M: male; yrs: years.

**Table 2:** Postoperative morphology of macula by SD-OCT.

	Group I number of eyes (%)	Group II number of eyes (%)	Group III number of eyes (%)	*p* I versus II	*p* II versus III	*p* I versus III
With foveal depression	10 (52.6%)	4 (20%)	11 (52.4%)	*0.047*	*0.048*	1.000
DONFL formation	0 (0%)	10 (50%)	7 (33.3%)	*0.001*	0.444	*0.019*
ERM recurrence	4 (21.1%)	0 (0%)	0 (0%)	*0.047*	1.000	*0.042*

DONFL: dissociated optic nerve fiber layer; ERM: epiretinal membrane; Group I: ERM removal without ILM peeling; Group II: ERM removal and whole-piece ILM peeling; Group III: ERM removal and ILM maculorrhexis; ILM: internal limiting membrane; SD-OCT: spectral-domain optical coherence tomography.

**Table 3:** Pre- and postoperative characteristics of ELM and EZ.

	Baseline		1 M		3 M		6 M		9 M		12 M	
	ELM(+)	EZ(+)	ELM(+)	EZ(+)	ELM(+)	EZ(+)	ELM(+)	EZ(+)	ELM(+)	EZ(+)	ELM(+)	EZ(+)
I (19)	11	12	11	12	15	14	19	17	19	19	19	19
II (20)	13	13	10	10	14	14	18	17	20	18	20	20
III (21)	13	14	13	14	16	16	21	19	21	21	21	21
*p* value												
I versus II	0.899	0.905	0.863	0.613	0.785	0.798	0.491	0.676	1	0.491	1	1
II versus III	0.837	0.88	0.651	0.444	0.925	0.925	0.447	0.954	1	0.447	1	1
I versus III	0.7986	0.816	0.796	0.816	0.835	0.855	1	0.916	1	1	1	1

ELM(+): preservation of external limiting membrane; EZ(+): preservation of ellipsoid zone; Group I: ERM removal without ILM peeling; Group II: ERM removal and whole-piece ILM peeling; Group III: ERM removal and ILM maculorrhexis; M: month.
